# Synthesis and biocompatibility of a biodegradable and functionalizable thermo-sensitive hydrogel

**DOI:** 10.1093/rb/rbv009

**Published:** 2015-08-20

**Authors:** Mantosh K. Sinha, Jin Gao, Chelsea E. T. Stowell, Yadong Wang

**Affiliations:** ^1^Department of Bioengineering and the McGowan Institute for Regenerative Medicine, University of Pittsburgh, Pittsburgh, PA 15219, USA;; ^2^Department of Surgery, University of Pittsburgh, Pittsburgh, PA 15260, USA;; ^3^Department of Chemical and Petroleum Engineering, Swanson School of Engineering, University of Pittsburgh, Pittsburgh, PA 15261, USA;; ^4^Department of Mechanical Engineering and Materials Science, University of Pittsburgh, Pittsburgh, PA 15261, USA;; ^5^Clinical Translational Science Institute, University of Pittsburgh School of Medicine, Pittsburgh, PA 15261, USA and; ^6^The McGowan Institute for Regenerative Medicine, University of Pittsburgh School of Medicine, Pittsburgh, PA 15261, USA

**Keywords:** biodegradable, thermoresponsive hydrogel, drug delivery, materials synthesize

## Abstract

Injectable thermal gels are a useful tool for drug delivery and tissue engineering. However, most thermal gels do not solidify rapidly at body temperature (37°C). We addressed this by synthesizing a thermo-sensitive, rapidly biodegrading hydrogel. Our hydrogel, poly(ethylene glycol)-*co*-poly(propanol serinate hexamethylene urethane) (EPSHU), is an ABA block copolymer comprising A, methoxy poly ethylene glycol group and B, poly (propanol L-serinate hexamethylene urethane). EPSHU was characterized by gel permeation chromatography for molecular weight and ^1^H NMR and Fourier transformed infrared for structure. Rheological studies measured the phase transition temperature. *In vitro* degradation in cholesterol esterase and in Dulbecco's phosphate buffered saline (DPBS) was tracked using the average molecular weight measured by gel permeation chromatography. LIVE/DEAD and resazurin reduction assays performed on NIH 3T3 fibroblasts exposed to EPSHU extracts demonstrated no cytotoxicity. Subcutaneous implantation into BALB/cJ mice indicated good biocompatibility *in vivo*. The biodegradability and biocompatibility of EPSHU together make it a promising candidate for drug delivery applications that demand carrier gel degradation within months.

## Introduction

Injectable, biodegradable reverse thermo-gels have ample applications in the field of bioengineering, especially if the gelling temperature is close to body temperature. Some potential uses include tissue engineering, gene delivery and drug release [1–3]. Injectable reverse thermo-gels possess both hydrophilic and hydrophobic domains. At low temperatures, the hydrophilic domains make the gels water soluble. Gelation is achieved by physical cross-linking of the hydrophobic domains upon temperature elevation without any chemical cross-linking [4–12].

Most researchers agree that a reverse thermo-gel intended for internal use should be in solution phase at room temperature and should form a physical gel at body temperature [13–19]. In addition, an ideal gel should have a defined degradation rate, to enable control of the release profile of a loaded drug. To guide cell-biomaterial interactions as well, the ideal gel would support functionalization with biologically active substances. With these three design objectives, we recently designed a reverse thermal gel, poly(ethylene glycol)-poly(serinol hexamethylene urethane) (ESHU), that has one free primary amine group on every repeating unit. This primary amine was successfully functionalized with the peptide IKVAVS (isoleucine–lysine–valine–alanine–valine–serine) to demonstrate the potential of cell adhesion control [20].

This reverse thermal gel is very useful due to its functionality, the facile control of the ratio of the hydrophilic and hydrophobic domains offered by its polyurethane chemistry and its potential as a biocompatible material [21–26]. Recently, we have applied ESHU for the sustained release of bevacizumab (Avastin), a drug used to treat age-related macular degeneration. However, ESHU degrades more slowly than our desired drug release window demands [27]. Here, we report a related gel that can also be functionalized but that possesses a faster degradation rate. The material is designed to be susceptible to hydrolytic degradation through ester bonds, logically forming carboxy- and hydroxy-terminated degradation products [28]. The polymer, poly(ethylene glycol)-*co*-poly(propanol serinate hexamethylene urethane) (EPSHU), is an ABA block copolymer comprising A, methoxy poly ethylene glycol (MPEG) group and B, poly (propanol L-serinate hexamethylene urethane). The degradation rate of EPSHU was determined in DPBS and in a solution of cholesterol esterase (CE). NIH 3T3 cytotoxicity assays and analysis of histological sections from subcutaneous injections in mice suggest good biocompatibility.

## Materials and Methods

### Materials

Boc-L-Serine and anhydrous N,N-dimethylformamide (DMF) were purchased from Acros. Hexamethylene diisocyanate (HDI) and N,N-dicyclohexyl carbodiimide (DCC) were bought from Sigma-Aldrich. 1-Ethyl-3-(3-dimethylaminopropyl)carbodiimide was obtained from Oakwood Chemical. 1,3-Propanediol, 4-(dimethyl amino) pyridine (DMAP) and poly(ethylene glycol) mono methyl ether (MPEG) were purchased from Alfa Aesar. The HPLC grade tetrahydrofuran (THF), dichloromethane (DCM), ethyl acetate (EtOAc), hexanes and anhydrous diethyl ether were purchased from Pharmco Aaper. The dialysis tubing was purchased from VWR (cutoff MW = 3.5 kD, Spectra/Por). The Dulbecco’s Modification of Eagle’s Medium (DMEM, both with and without L-glutamine), the penicillin G, the streptomycin sulfate, the amphotericin B and the L-glutamine were all purchased from Cellgro. Fetal bovine serum (FBS) was purchased from Life Technologies. CE was obtained from Worthington Biochemical Corporation. The LIVE/DEAD viability/cytotoxicity kit was purchased from Molecular Probes. The Cell-Titer Blue kit was purchased from Promega. The rat monoclonal antibody ED1 (anti-CD68) was purchased from Abcam. The goat anti-rat secondary antibody conjugated with Alexa594 was purchased from Life Technologies.

### Instruments

Isolera One Biotage chromatography was used for purification of the intermediates. Nuclear magnetic resonance (NMR) spectra were recorded on a Bruker Avance 600 MHz instrument. Fourier transformed infrared (FTIR) spectra were recorded on a Thermo Nicolet iS10 spectrometer equipped with a diamond Smart iTR. The molecular weight was recorded via gel permeation chromatography (GPC) on a Viscotek GPCmax VE2001 with a Viscotek 270 dual detector (differential refractive index and right angle light scattering), using an SDV GPC analytical column (1000Å 5µ, posportiy 8 × 300 mm). Rheological measurements were recorded on a thermostatted oscillating rheometer (PHYSICA MCR301, Anton Paar) equipped with a 5 cm steel cone (1°). A Countess hemocytometer was used as an automated cell counter during passaging. Fluorescent and brightfield images were acquired on a Nikon ECLIPSE Ti equipped with a Qimaging RETIGA-SRV digital camera. The CellTiter-Blue assay fluorescence measurements were performed on a microplate reader using Gen5 software (SYNERGY Mx, BioTek).

### General methods

All commercially available reagents and solvents were used without further purification. All reactions were carried out under dry nitrogen or in a glove box and in oven-dried glassware. TLC was performed on glass sheets precoated with silica gel (Merck, Kieselgel 60, F_254_). Column chromatographic separations were performed on silica gel Silicycle cartage. ^1^H and ^13^C NMR spectra were measured in CDCl_3_, and neat samples were used for FTIR. NMR abbreviations used are s = singlet, brs = broad singlet, d = doublet, dd = double doublet, t = triplet and m = multiplet. Data in parentheses are given in the following order: multiplicity, number of protons and coupling constants in Hz.

### Synthesis of EPSHU

**Scheme 1.**
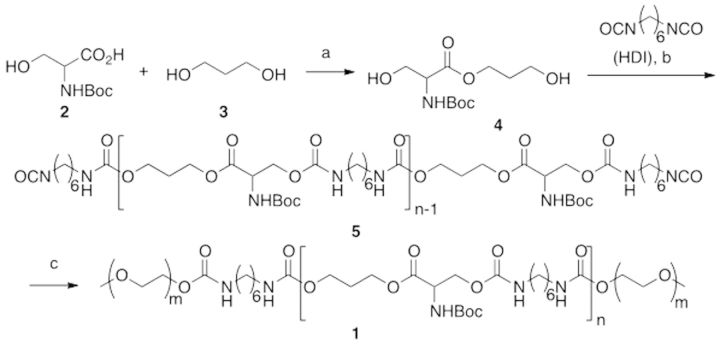
Synthesis of EPSHU: (a) DCC, DMAP, 0 − rt, 24 h, 88%; (b) 90 °C, 2 h, then HDI in excess, DMF, 90 °C, 16 h; (c) MPEG 550, DMF, 90 °C, 24 h. 151x72mm (100 x 100 DPI).

The synthesis is summarized in Scheme 1. Bolded numbers after compound names in the text refer to structures in the reaction scheme.

#### 3-Propanol L-serinate (4)

DCC (5.15 g, 25 mmol) was added to a stirring solution of 1,3-propanediol (**3**) (19 g, 250 mmol) in THF/DCM (1:1; 500 ml) at ambient temperature followed by addition of DMAP (122 mg, 2.5 mmol). The mixture was stirred at the same temperature for 30 min, and then NBoc L-Serine (**2**) (5.13 g, 25 mmol) in THF/DCM (1:1; 30 ml) was added drop-wise using a syringe pump over 4 h at 0°C. The resulting mixture was stirred for 48 h, during which time it was allowed to rise to ambient temperature. The reaction mixture was concentrated under reduced pressure, and the residue was dissolved in EtOAc. After addition of saturated NaHCO_3_ solution, the resulting mixture was extracted with EtOAc. The organic phase was washed with brine and dried in Na_2_SO_4_. Solvents were evaporated under reduced pressure after filtration to obtain the crude product. This crude residue was purified by column chromatography using gradient elution EtOAc/Hexanes (0:100–85:15) over 30 min to yield **4** (88%) as a colorless viscous liquid. ^1^H NMR (600 MHz, CDCl_3_) of intermediate **4** showed peaks of the following intensities, multiplicities and shifts (ppm), which were assigned to the listed protons: δ: 1.47 (s, 9H), 1.89–1.85 (m, 2H), 3.40 (brs, 2H), 3.68 (t, *J* = 6 Hz, 2H), 3.81 (dd, *J* = 11.4, 3.6 Hz, 1H), 3.97 (d, *J* = 10.8 Hz, 1H), 4.24–4.20 (m, 1H), 4.31 (s, 1H), 4.40–4.36 (m, 1H), 5.66 (d, *J* = 7.2 Hz, 1H) (full spectrum in Supplementary Fig. 1S); ^13^C NMR (150 MHz) δ: 28.3, 31.1, 55.8, 59.0, 62.7, 63.1, 80.2, 155.8, 171.3 (full spectrum in Supplementary Fig. 2S).

#### Poly (3-propanol L-serinate hexamethylene urethane) (5)

Equimolar amounts of 3-propanol L-serinate (**4**) (0.5 g, 1.9 mmol) and 1,6-diisocyanatohexane (HDI) (320 mg, 1.9 mmol) were mixed in a 25 ml round bottom flask with a stir bar in a glove box filled with nitrogen. The flask was sealed, transferred out of the glove box and connected to a Schlenk line. The reaction mixture was heated to 90°C under a nitrogen atmosphere for 130 min. After that, DMF (15 ml) and an excess of HDI (640 mg, 3.8 mmol) were added to the reaction, and the resulting mixture was heated for an additional 16 h, all still at 90°C. The reaction mixture was transferred to an excess of anhydrous diethyl ether (250 ml) and stirred under nitrogen. As stirring proceeded, the more viscous reaction mixture sank to the bottom of the vessel, leaving a transparent ether layer on top. The ether phase was decanted carefully, and the residue was dried under vacuum at room temperature for 30 min. Then, ether was added to the residue again, and the above procedure was repeated to further purify the intermediate. Afterward, the resulting residue (**5**) was dried under vacuum at room temperature. ^1^H NMR (600 MHz, CDCl_3_) of intermediate **5** showed peaks of the following intensities, multiplicities and shifts (ppm), which were assigned to the listed protons (full spectrum in Supplementary Fig. 4S): δ: 4.95–5.75 [m, 3H, NH], 4.05–4.65 [m, 7H, –CO_2_–CH_2_–CH(NHBoc) –CO_2_–CH_2_CH_2_CH_2_–OCO–]; 3.05–3.35 [m, 4H, –NH–CH_2_(CH_2_)_4_CH_2_–NH–]; 1.95–2.1 [m, 2H, –CO2CH_2_–CH_2_–CH_2_OCO–]; 1.45–1.63 [m, 4H, –NHCH_2_–CH_2_– (CH_2_)_2_– CH_2_–CH_2_NH–]; 1.43–1.45 (m, 9H, –OC(CH_3_)_3_]; 1.38–1.41 [m, 4H, –NH(CH_2_)_2_–CH_2_–CH_2_–(CH_2_)_2_NH–].

#### Poly(ethylene glycol)-co-poly(propanol serinate hexamethylene urethane) (1)

Intermediate **5** and MPEG (4 g, Mw: 550) were dissolved in DMF (4 ml) in a 25 ml round-bottom flask. The resulting mixture was heated to 90°C for 24 h under nitrogen. Then the reaction mixture was transferred to excess anhydrous ether (250 ml) and purified in the same way as described previously. After drying, the polymer was further purified by dialysis (cut off Mw: 3500) in water at 4°C. The dialyzed solution was freeze-dried to give a transparent EPSHU (yield: 92% in two steps).

### *In vitro* degradation

The *in vitro* degradation of EPSHU was carried out according to previously described procedures [20, 29]. CE (1960 units/gm DW) was used as a model esterase. The supplier defined one unit of CE as the mass of enzyme required to produce 1.0 µmol of cholesterol from cholesteryl oleate each minute. (Readers comparing this assay to that described for ESHU in Ref. [20] should note that that CE supplier defined one unit of CE as 1/1000th of the unit this supplier used. After unit conversion, one will find the assay conditions identical.) EPSHU was dissolved in 5% (wt/v) in 5 ml DPBS containing 0.5% NaN_3_ solution and incubated with 0.4 units/ml CE at 37°C on an orbital shaker at 60 rpm; 0.2 ml of freshly prepared CE (2 u/ml) was added to the polymer solution every 3 days to replenish the pool of active CE [29]. The degradation in DPBS was performed identically but without the enzyme. At each time point, 1 ml of EPSHU in solution was taken from each system, lyophilized for 72 h and stored at −20°C. After thawing, the partially degraded EPSHU was dissolved in THF and filtered at 0.2 µm before its average MW was measured by GPC.

### LIVE/DEAD assay

The gel extract cytotoxicity testing was performed according to ISO 10993-5 and the instructions of the LIVE/DEAD assay kit manufacturers [30]. NIH 3T3s were cultured at 37°C and 5% CO_2_ in DMEM supplemented with 10% FBS, 100 u/ml penicillin G, 100 µg/ml streptomycin sulfate and 0.25 µg/ml amphotericin B. Cells were subcultured at 50–70% confluency, approximately every 3 days and used at least two passages after thawing from liquid nitrogen storage; 1.1 ml of EPSHU dissolved in DPBS (20%; wt/v) was filtered at 0.2 µm and incubated at 37°C overnight. After gelation, 1.1 ml of fresh culture media was added to the vial. The system was incubated for an additional 24 h to produce a 1X-strength culture media extraction of the solid gel. Concurrently, 3T3s were seeded into a 96-well plate at 6000 cells/well. After allowing the cells 24 h to attach, the culture media was exchanged for 150 µl of freshly produced extraction medium. Plain culture media that had incubated alongside the extract was given to control wells. The cells were incubated at 37°C and 5% CO_2_ for 24 h. Cells were then washed with DPBS, and a 100 µl solution of DPBS with 10 µmol calcein AM and 10 µmol ethidium homodimer-1 (LIVE/DEAD assay kit) was applied. Fluorescent images in the FITC (calcein) and TRITC (ethidium) channels were captured after at least 30 min of incubation at RT. The numbers of live and dead cells were counted using the Nikon Ti Eclipse image analysis software in three randomly chosen 100X fields in each of three wells per treatment group.

### CellTiter-blue toxicity assay

*In vitro* cytotoxicity was assessed by means of the CellTiter-Blue assay. NIH 3T3s were cultured at 37°C and 5% CO_2_ in DMEM (supplied without glutamine) supplemented with 10% FBS, 2 mM L-glutamine, 100 µg/ml penicillin G and 100 µg/ml streptomycin sulfate. Culture conditions were otherwise identical to those in the LIVE/DEAD assay; 1.2 and 0.625 ml aliquots of EPSHU dissolved in DPBS (20%; wt/v) were filtered at 0.2 µm and incubated at 37°C overnight. After gelation, 1.2 and 1.25 ml of fresh culture media was added to the two vials. The system was incubated for an additional 24 h to produce 1X and 0.5X-strength extractions, respectively. NIH 3T3 fibroblasts (3200 cells/well) were grown over 24 h in a 96-well plate. Cells were washed with fresh medium before the extracts were applied. Plain culture media was given to control wells. Cells were cultured for 1, 3 and 5 days (*n* = 3 per group and timepoint) at 37°C. Cells were then treated with 20 µl of CellTiter-Blue (resazurin) solution and incubated for an additional 4 h at 37°C. Fluorescence was measured in triplicate for each well at 560 nm/590 nm excitation/emission.

### *In* *vivo* biocompatibility

Solid EPSHU was sterilized by UV for 1 h in a biological cabinet, then dissolved at 20% (wt/v) solution in sterile saline and stored at 4°C before injection. Under sterile conditions, 8-week-old male BALB/cJ mice weighing 22–25 g were injected subcutaneously with 100 µl of EPSHU solution (*n* = 3) in the lower right back after brief anesthestization with 2–5% isoflurane. Saline (100 µl) was injected in the lower left back of each mouse as a control. Animals were cared for in compliance with a protocol approved by the Committee on Animal Care of the University of Pittsburgh, following National Institutes of Health guidelines for the care and use of laboratory animals.

Mice were euthanized and samples harvested at 3, 14 and 28 days after injection. Tissues were fixed in 10% neutral buffered formalin for 15 min before histological analysis. The control tissues were similarly harvested and fixed at the same time points. The tissues were soaked in 30% sucrose and embedded in O.C.T. Six-micrometer-thick sections were cut along each sample. Sections were stained using standard protocols for hematoxylin and eosin (H&E) and Masson's trichrome stain (MTS). The inflammatory response to each sample was assessed by immunohistochemical analysis: briefly, 6 -μm-thick sections of the tissues were dried and fixed in histology-grade absolute ethanol for 15 min, air dried and incubated with rat monoclonal ED1 (1:200) for macrophage and monocyte identification. The slides were then incubated with a goat anti-rat-Alexa 594 (1:400) for 1 h. Stained sections were analyzed for the density of newly recruited macrophages. Five to 10 200X magnification images were obtained for each specimen. The number of CD68-positive cells was quantified using NIH Image J software.

### Statistical analysis

The data are expressed as mean ± SD. Per-well percent viabilities in the LIVE/DEAD assay were compared by an unpaired Student’s *t*-test assuming equal variances (α = 0.05). CellTiter-Blue assay viabilites were compared within each timepoint by one-way ANOVAs, which, if significant (*P* < 0.05), were followed by sets of t-tests with a Bonferroni correction (adjusted *P < 0.017). Student's *t*-test was used to determine whether the numbers of CD68-positive cells in each group differed significantly from each other (**P < 0.01.)

## Results and Discussion

### Synthesis and characterizations

**Figure 1.**
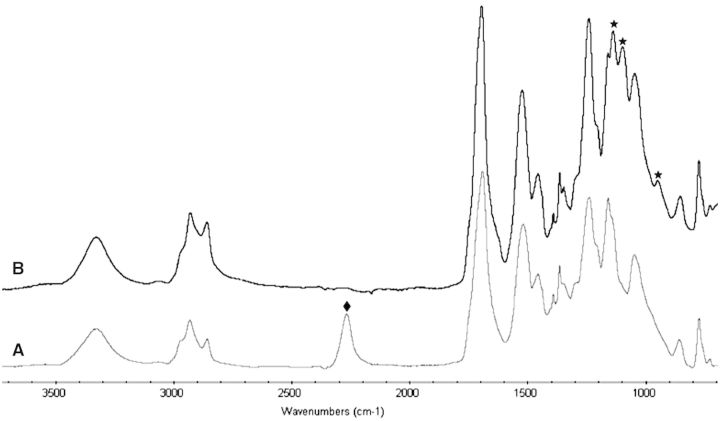
FTIR spectra of (**A**) intermediate 5 and (**B**) EPSHU (1): after the PEGylation, the reactive isocyanate groups of intermediate 5 at 2267 cm^−1^ (diamonds) completely disappeared and peaks at 975, 1099 and 1139 cm^−1^ (stars) appeared.

3-propanol L-serinate (**4**) was synthesized from N-Boc serine (**2**) and an excess of 1,3-propanediol (**3**) by using DCC coupling. The monomer (**4)** was characterized by ^1^H NMR, ^13^C NMR, FTIR and LCMS, as shown in the Supplementary Material. EPSHU was then polymerized in the two basic steps shown in Scheme 1. The first step was the polymerization of **4** and HDI, followed by the addition of an excess of HDI. The excess ensured isocyanate groups on both ends as in **5**. This intermediate was characterized by the appearance of a new peak at 2267 cm^−1^ (Fig. 1).

In the second step, this intermediate was treated with MPEG (550) to produce EPSHU (**1)**. As seen in the FTIR spectra in Fig. 1A and B, the peak at 2267 cm^−^^1^ for isocyanate (−N=C=O) disappeared, and new peaks appeared at 975, 1099 and 1139 cm^−^^1^. These changes indicate the MPEGylation of both isocyanate end groups of **5**. Peaks at 1696, 1525 and 1242 cm^−^^1^ were assigned as carbonyl (both amide and ester), NH and the urethane C−O−C.

**Figure 2.**
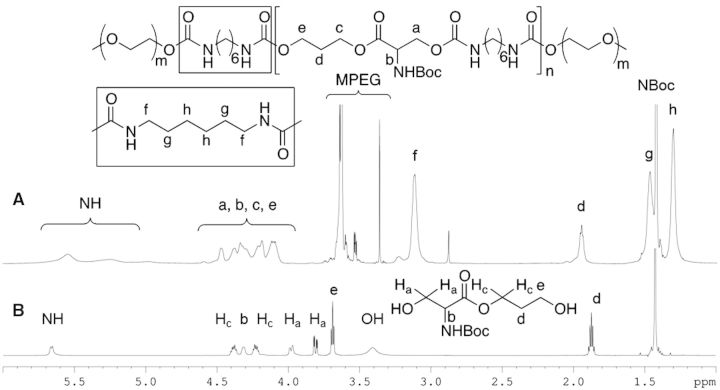
^1^H NMR: comparison of EPSHU (1) (**A**) and serinate (4) (**B**).

The ^1^H NMR of serinate (**4**) and EPSHU (**1**) were compared for further analysis. The proton peak at 3.69 (d), 3.81 (H_a_) and 3.97 (H_a_) ppm (Fig. 2A) had moved downfield after forming an amide bond with the isocyanate. The protons of MPEG were assigned at 3.61 and 3.54–3.66 ppm. Protons from HDI (f, g and h) appeared at 2.78, 1.47 and 1.3 ppm, respectively (Fig. 2B).

The molecular weight of EPSHU was measured by GPC using poly (ethylene glycol) as a standard in THF. The molecular weight of EPSHU was approximately 13 000 with relatively low PDI (1.88) (Supplementary Fig. 7S).

**Figure 3.**
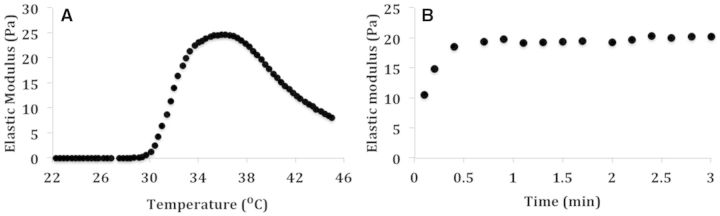
The elastic modulus of EPSHU 10% (wt/v). (**A**) Temperature sweep was recorded over the range 22–45°C. (**B**) Time sweep was recorded at 37°C for 3 min.

The first design criterion for our ideal injectable gel was that it gel quickly at body temperature but remain fluidic at room temperature. The rheometer measurements of elastic modulus over temperature and time sweeps are shown in Fig. 3. For this study, EPSHU (10% wt/v) was dissolved in DPBS at low temperature (4°C). It was a transparent solution at 4°C but transformed into an opalescent hydrogel at physiological temperature (37°C). There were no significant changes in elastic modulus below 30°C (Fig. 3A). The sol–gel transition was achieved at 35–37°C. Further heating above 38°C led to a decrease in elastic modulus. The time sweep was measured to estimate the speed of gel formation at body temperature (37°C). It took less than a minute for EPSHU (10% wt/v) to form a gel (Fig. 3B). These results hold promise for internal injection applications.

### *In* *vitro* degradation

An appropriate degradation rate was our second design criterion for an ideal gel. The degradation assay was performed identically to that done on previously synthesized ESHU in Ref. [20] to allow direct comparison of the two gels. By Day 45, the molecular weight of ESHU had decreased by 1.9% in DPBS and by 20.2% in CE. We hypothesized that the substitution of the more hydrolysis-susceptible ester bonds in EPSHU for the amide bonds in ESHU would accelerate degradation.

**Figure 4.**
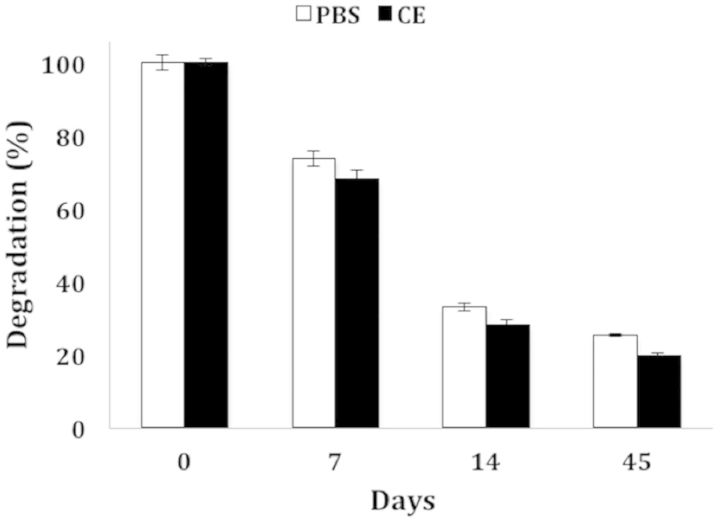
Degradation of EPSHU in DPBS and in CE solution, expressed as percent of average MW at Day 0. The degradation rate in CE solution was barely faster than in DPBS alone. Data are presented as mean ± SD (*n* = 3).

We measured the average molecular weight of EPSHU dissolved in 37°C DPBS and CE solutions over 6 weeks. (The GPC molecular weight distributions are available in Supplementary Fig. 8S.) By Days 7, 14 and 45, the molecular weight of EPSHU decreased to 26.4, 67.2 and 75% of its original value in DPBS and 32.2, 72 and 80.7% in CE, as shown in Fig. 4. This degradation profile was substantially faster than that of ESHU. Because a polyester hydrolyzes quickly in a DPBS solution [31], there was not much difference in the degradation rate of EPSHU in DPBS and the CE solutions.

### Biocompatibility

#### LIVE/DEAD assay

**Figure 5.**
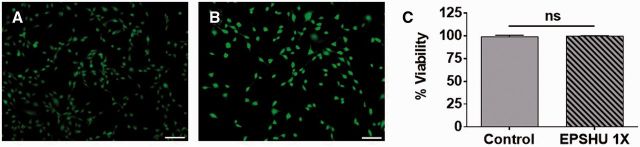
NIH 3T3 LIVE/DEAD assay. (**A**) Culture media control. (**B**) 1X EPSHU extract. Green indicates a live cell, and red indicates a dead cell. Color images are available online. All cells are green (light gray in the black and white images), no red cells were detected. Bar is 100 µm. (**C**) Percent viability in 3T3s exposed to control media or 1X EPSHU extract. Data are presented as mean ± SD (*n* = 3).

The LIVE/DEAD assay was used as a qualitative evaluation of cytotoxicity. 3T3s cells were cultured with EPSHU extract *in vitro* to observe the cell viability. After 24 h of incubation with EPSHU extract or with control culture media, 3T3s in both groups displayed a healthy fibroblast morphology (Fig. 5A and B). There was no significant difference in the percent viabilities; nearly all cells in both groups were metabolically active and intact (Fig. 5C).

#### CellTiter-blue assay

**Figure 6.**
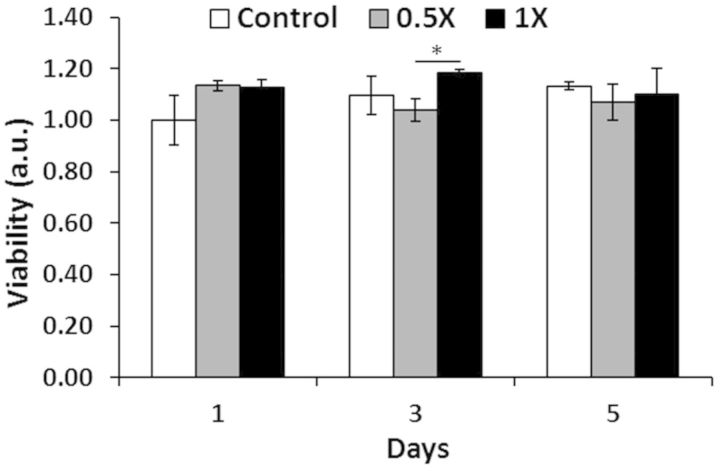
CellTiter-Blue assay: effects of 0.5X and 1X strength EPSHU extracts on NIH 3T3 metabolic activity after 1, 3 and 5 days of exposure. Data are presented as mean ± SD (*n* = 3), normalized to the control (culture media) absorbance at Day 1. *P < 0.05.

The CellTiter-Blue assay was performed to more rigorously quantify cytotoxicity. This assay is a fluorescent analog of the well-known MTT assay for cell metabolic activity and number. NIH 3T3 fibroblasts were cultured with 1X and 0.5X strength extractions of gelled EPSHU for 1, 3 and 5 days. As shown in Fig. 6, there were no sustained significant differences between EPSHU groups and control over time. These results, along with those of the LIVE/DEAD assay, indicate the gel possesses minimal cytotoxicity.

#### *In vivo* biocompatibility

To examine the biocompatibility of EPSHU *in vivo*, 100 µl of EPSHU dissolved in saline (20% w/v) was subcutaneously injected into mice. Immediately after injection, the EPSHU solutions formed visible hydrogels under the skin that maintained their shape over the entire 28-day experimental period without showing any signs of inflammation.

**Figure 7.**
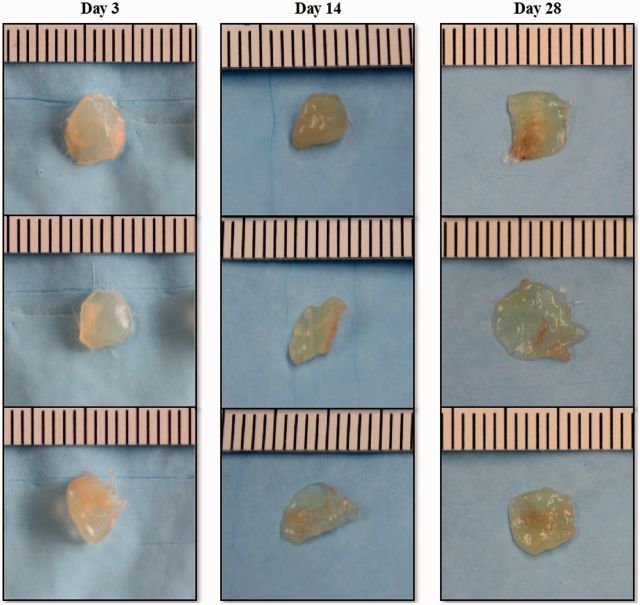
EPSHU gels explanted from mice after 3, 14 and 28 days (*n* = 3).

The implants were excised 3, 14 and 28 days after injection (Fig. 7). It was extremely difficult to separate the pure polymer from the biological components of the explant, so the precise *in vivo* degradation profile of EPSHU could not be determined in this study. However, the shrinking dimensions of the explants indicate that the polymer degrades rapidly *in vivo*.

**Figure 8.**
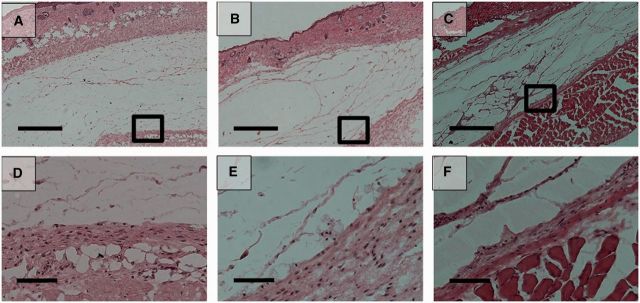
Hematoxylin and eosin (H&E) -stained histological sections of the tissue adjacent to the sites of subcutaneous injection of 100 μl of 20% (wt/v) EPSHU into BALB/cJ mice. The EPSHU degraded gradually. Cavities with cell infiltrates had formed inside the gel at Day 3 (**A** and **D**), Day 14 (**B** and **E**) and Day 28 (**C** and **F**) after injection. (A–C) Low magnification of images (40×, scale bar = 500 μm). The rectangular frames indicate the fields chosen for capture at higher magnifications (D–F, 200×, scale bar = 100 μm).

**Figure 9.**
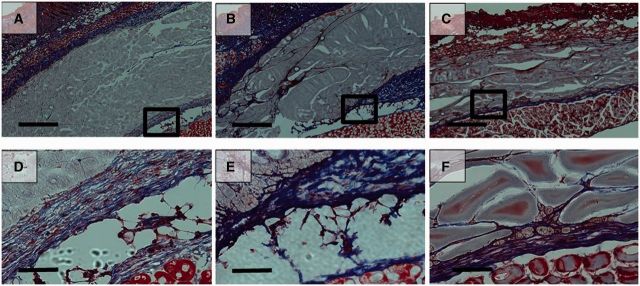
MTS histological sections of the tissue adjacent to the sites of subcutaneous injection of 100 μl of 20% (wt/v) EPSHU into BALB/cJ mice. MTS confirmed that collagen deposition around the polymer (blue fibers, color images are available in downloadable pdf online.) was minimal at Day 3 (**A** and **D**), Day 14 (**B** and **E**) and Day 28 (**C** and **F**). (A–C) Low magnification of images (40×, scale bar = 500 μm). The rectangular frames indicate the fields chosen for capture at higher magnifications (D–F, 200×, scale bar = 100 μm).

**Figure 10.**
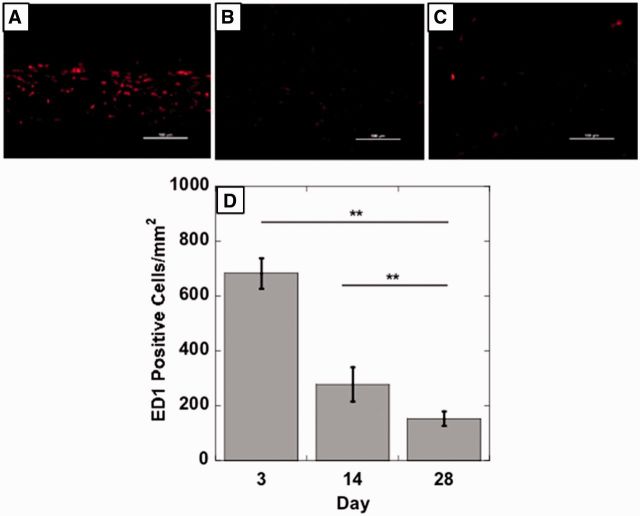
Representative photomicrographs (200×, scale bar = 100 μm) of injection sites immunohistochemically stained with ED1, an antibody against the monocyte/macrophage marker CD68. Tissues were harvested on (**A**) Day 3, (**B**) Day 14 and (**C**) Day 28 post-injection. (**D**) The number of ED1-positive cells decreased with time, indicating a reduction in the inflammatory response. Images from 5 random areas around the injection sites were used for quantification at each time point. Data are shown as mean ± SD (*n* > 5). **P < 0.001.

Histological images showed a gradual degradation of EPSHU at Days 3, 14 and 28 after injection (Figs 8 and 9). At Day 28, cavities had formed inside the material with phagocytic inflammatory infiltrates (Fig. 8C and F). Cells had also infiltrated into the polymer and begun to remodel the gel at Day 28. H&E staining revealed the presence of mild inflammatory infiltrates around the polymer. MTS staining indicated that collagen (blue fibers) deposition surrounding the polymer residue was loose and thicker at Days 3 and 14 (Fig. 8A, B, D and E) than it was at Day 28 (Fig. 9C and F), when it became minimal. The EPSHU caused little fibrosis, and muscle degeneration was observed at the three time points. By Day 3 after injection, the tissue adjacent to the gel and an inner area close to the gel contained mostly inflammatory cells (Fig. 9A). At Day 14, there was a marked decrease in newly recruited macrophages compared with Day 3 (Fig. 10A, B and D). The inflammatory cells inside the gel and connective tissue beneath were very minimal at Day 28 (Fig. 10C and D). The number of CD68-positive cells was significantly decreased compared with both Days 3 and 14, which indicated that an inflammation was resolved after 28 days injection.

## Conclusion

We developed a biodegradable thermo-sensitive hydrogel that demonstrates good biocompatibility *in vitro* and *in vivo*. EPSHU showed similar biocompatibility to and retains the functionalization capacity of our previous gel, ESHU, but degrades more quickly. We believe that this injectable reverse thermal gel could be very useful in drug delivery and tissue engineering applications where month-scale degradation is needed.

## Supplementary Material

Supplementary Fig. 1S
