# Supplementary material for: Synthesis and biocompatibility of a biodegradable and functionalizable thermo-sensitive hydrogel
Source: Regen Biomater. 2015 Aug 20;2(3):177–85. doi: 10.1093/rb/rbv009 (PMC4669011; doi:10.1093/rb/rbv009)
Supplement: Supplementary Fig. 1S [file rb_rbv009_index.html]

Supplementary Data | Regenerative Biomaterials

## Supplementary Data

files

- Supplementary Data - docx file
