# Supplementary material for: Synthesis and biocompatibility of a biodegradable and functionalizable thermo-sensitive hydrogel
Source: Regen Biomater. 2015 Aug 20;2(3):177–85. doi: 10.1093/rb/rbv009 (PMC4669011; doi:10.1093/rb/rbv009)
Supplement: Supplementary Fig. 1S [file Supporting_Information.docx]

Supporting Information

**Synthesis and Biocompatibility of a Biodegradable and Functionalizable Thermo-Sensitive Hydrogel**

**Mantosh K. Sinha^1^, Jin Gao^1^, Chelsea E. Stowell^1^, Yadong Wang^1-6*^**

^1^Department of Bioengineering and the McGowan Institute for Regenerative Medicine, University of Pittsburgh, Pittsburgh, PA 15219, USA

^2^Department of Surgery, University of Pittsburgh, Pittsburgh, PA, USA 15260

^3^Department of Chemical and Petroleum Engineering, Swanson School of Engineering, University of Pittsburgh, Pittsburgh, PA, USA 15261

^4^Department of Mechanical Engineering and Materials Science, University of Pittsburgh, Pittsburgh, PA, USA 15261

^5^Clinical Translational Science Institute, University of Pittsburgh School of Medicine, Pittsburgh, PA, USA 15261

^6^The McGowan Institute for Regenerative Medicine, University of Pittsburgh School of Medicine, Pittsburgh, PA, USA 15219

*Correspondence address. Department of Bioengineering, University of Pittsburgh, Pittsburgh, PA 15261, USA. Tel.: +1 412 624 7196; fax: +1 412 383 8788. E-mail address: [yaw20@pitt.edu](mailto:yaw20@pitt.edu) (Y. Wang).

**List of Contents:**

| 1. | Fig. **1S**: ^1^H NMR of **4** ----------------------------------------------------------------- | S-2 |
| --- | --- | --- |
| 2. | Fig. **2S**: ^13^C NMR of **4** ---------------------------------------------------------------- | S-3 |
| 3. | Fig. **3S**: FTIR of **4** --------------------------------------------------------------------- | S-4 |
| 4. | Fig. **4S**: ^1^H NMR of **5** ----------------------------------------------------------------- | S-5 |
| 5. | Fig. **5S**: FTIR of **5** --------------------------------------------------------------------- | S-6 |
| 6. | Fig. **6S**: ^1^H NMR of **1** ----------------------------------------------------------------- | S-7 |
| 7. | Fig. **7S**: ^13^C NMR of **1** ---------------------------------------------------------------- | S-8 |
| 6. | Fig. **8S**: FTIR of **1** --------------------------------------------------------------------- | S-9 |
| 7. | Fig. **9S**: GPC spectra for molecular weight of **1** ----------------------------------- | S-10 |
| 8. | Fig. **10S**: GPC spectra for degradation of EPSHU in day 0, 7, 14 and 45 ----- | S-11 |

**
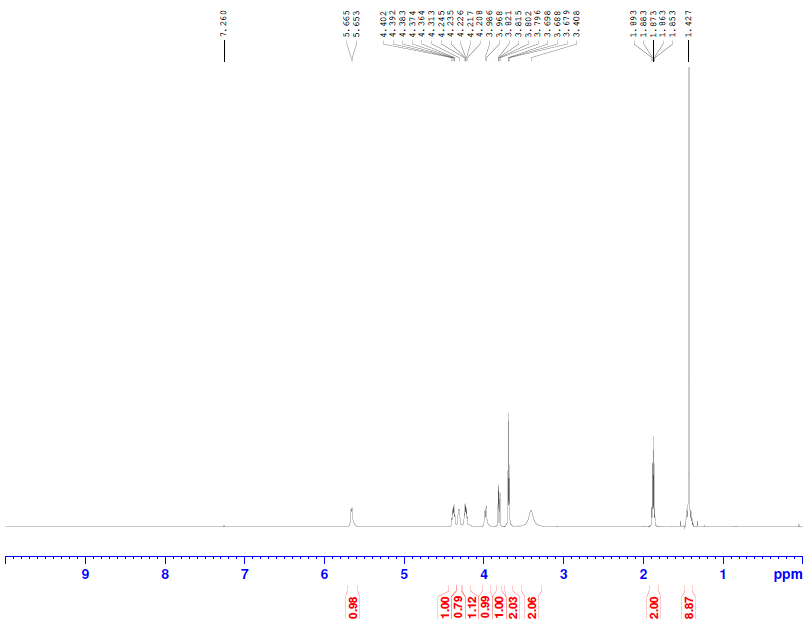
Fig. 1S:** ^1^H NMR of **4** in CDCl_3_.

**Fig. 2S:** ^13^C NMR of **4** in CDCl_3_
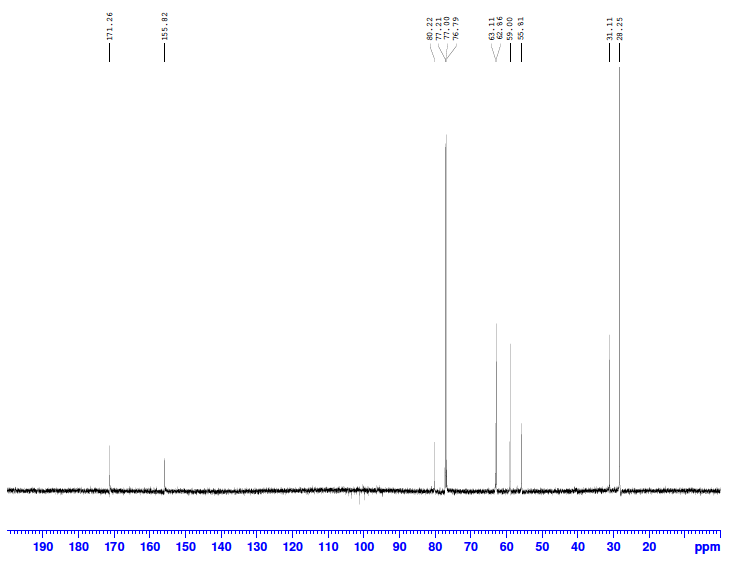
.


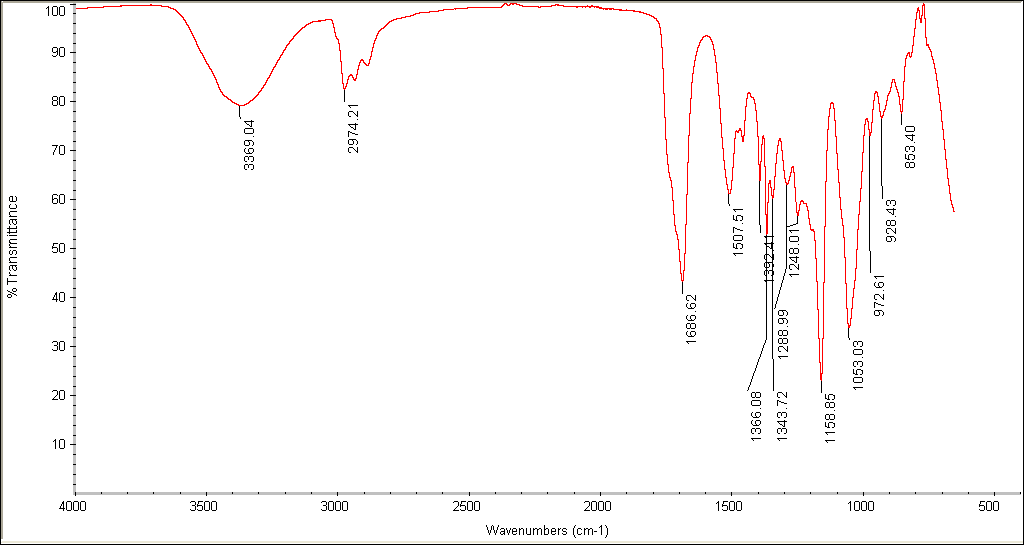


**Fig. 3S:** FTIR of **4** neat.

**Fig. 4S:** ^1^H NMR of **5** in CDCl_3_
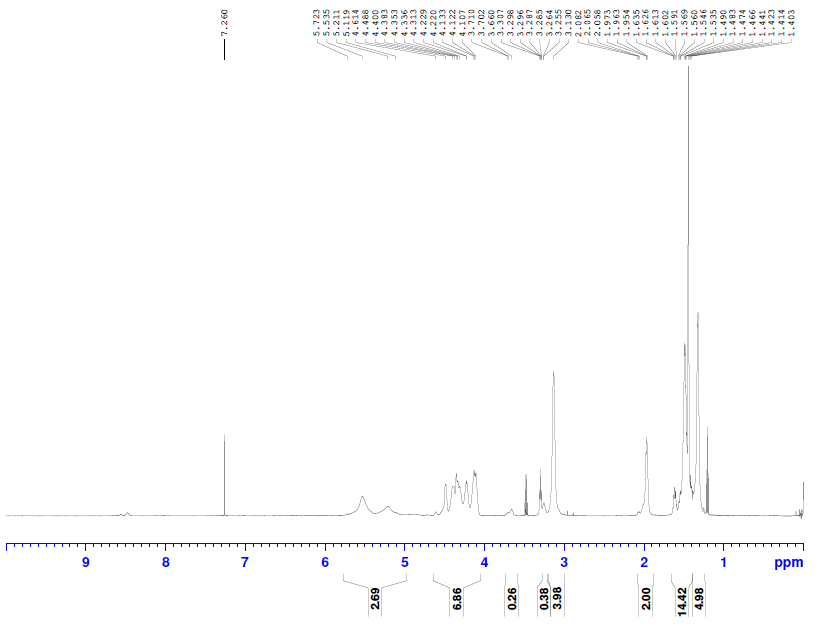
.


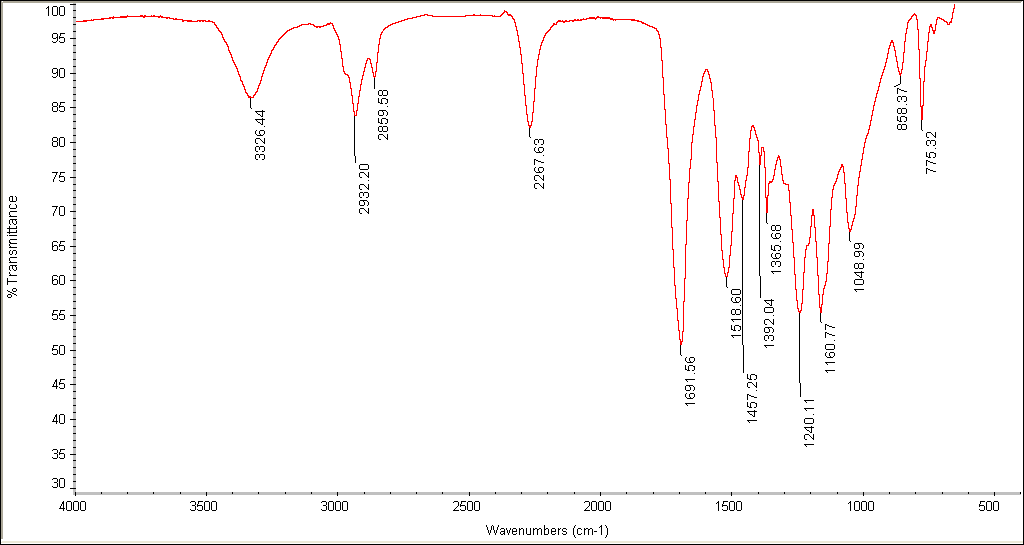


**Fig. 5S:** FTIR of **5** neat.

**Fig. 6S:** ^1^H NMR of **1** in CDCl_3_
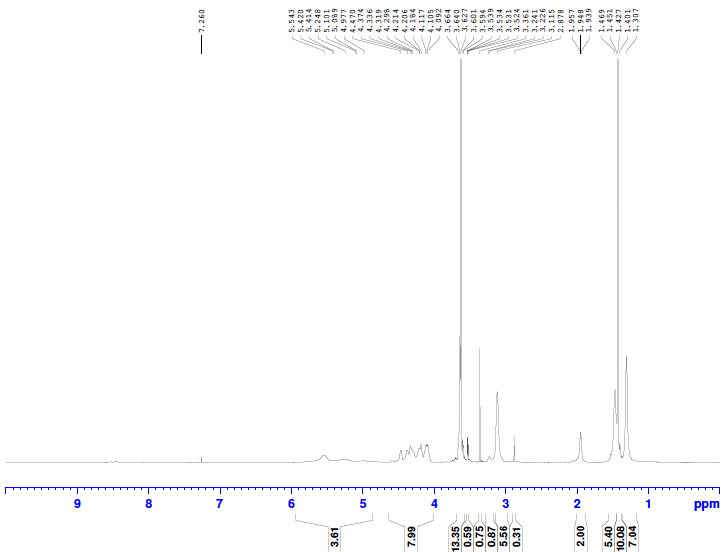


**Fig. 7S:** ^13^C NMR of **1** in CDCl_3_ _
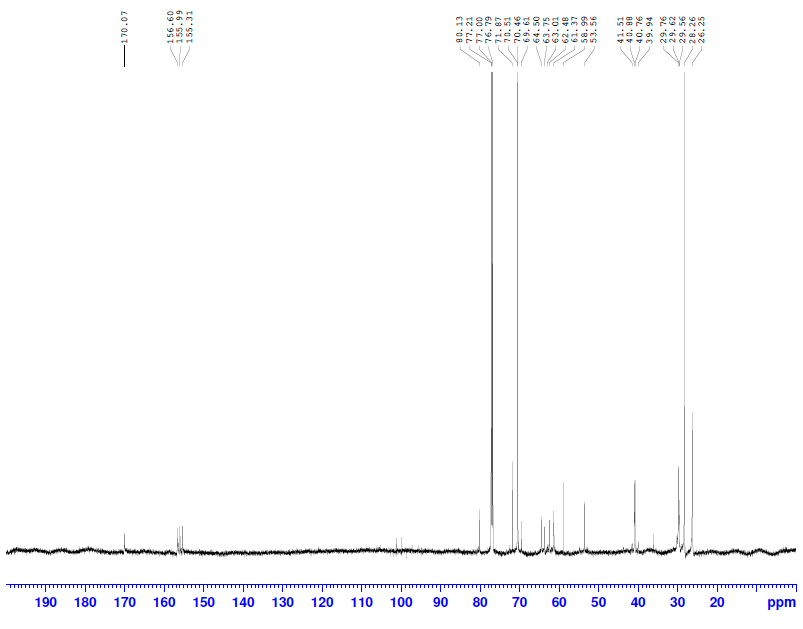
_

**
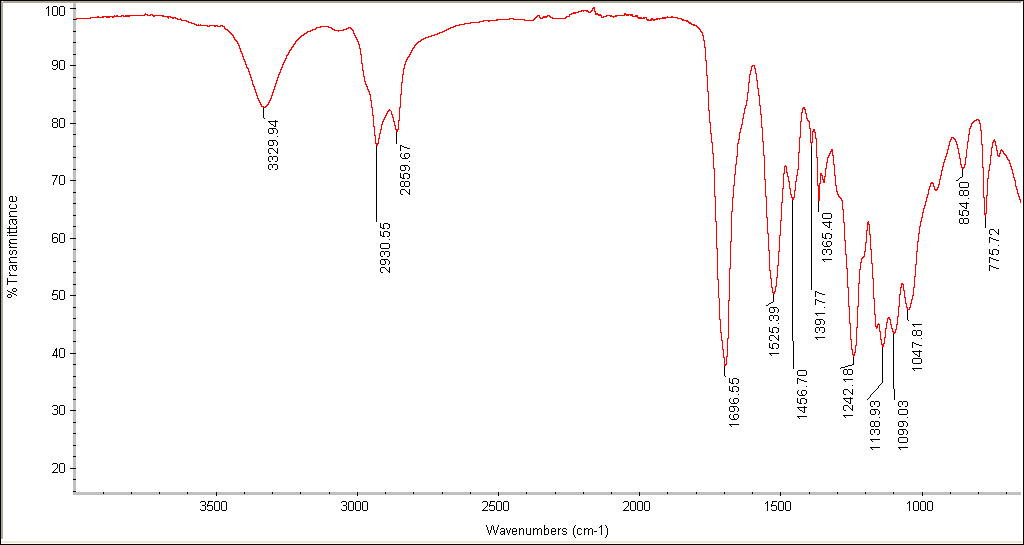
Fig. 8S:** FTIR of **1** neat.


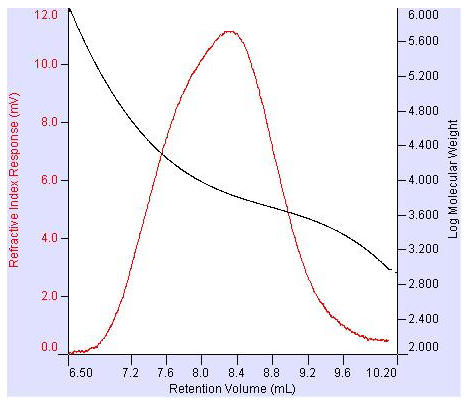

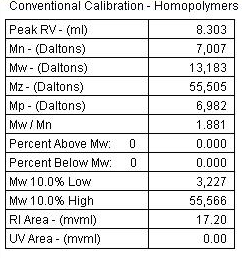
 **Fig. 9S:** GPC spectra for molecular weight of **1** (THF 1 ml/ min)
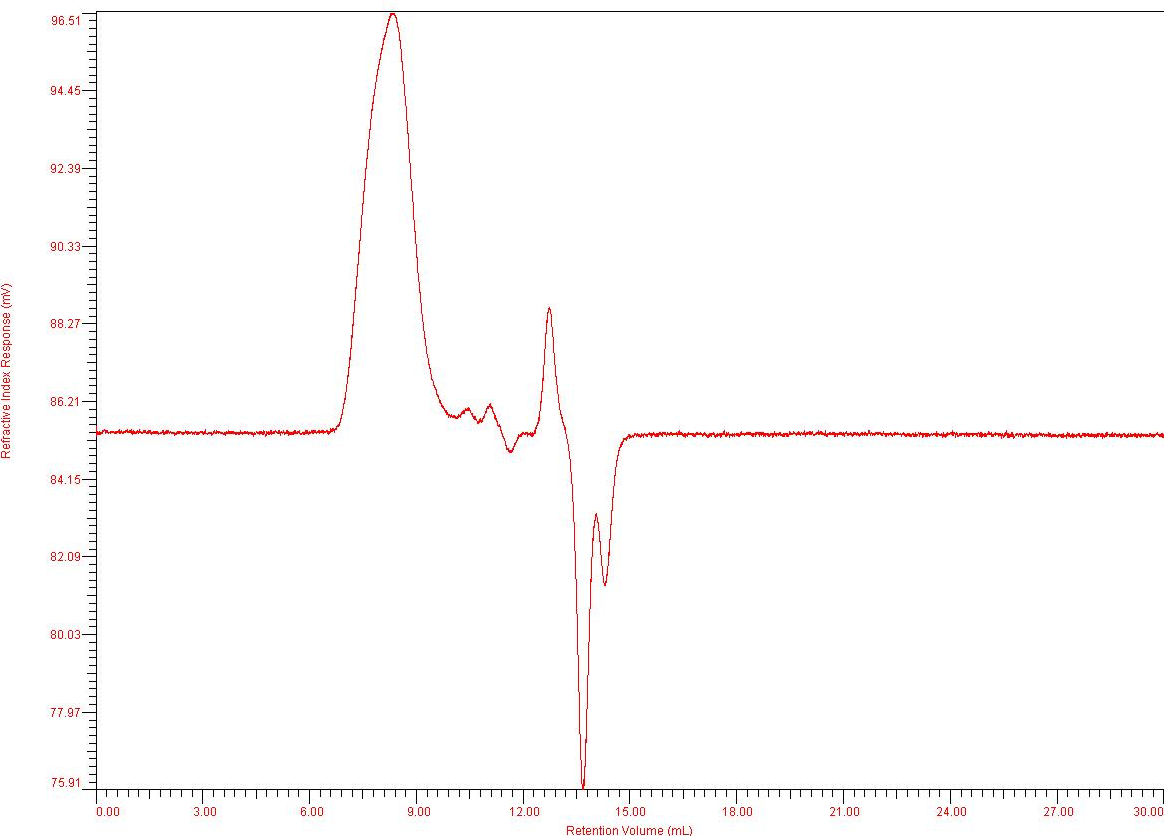
.


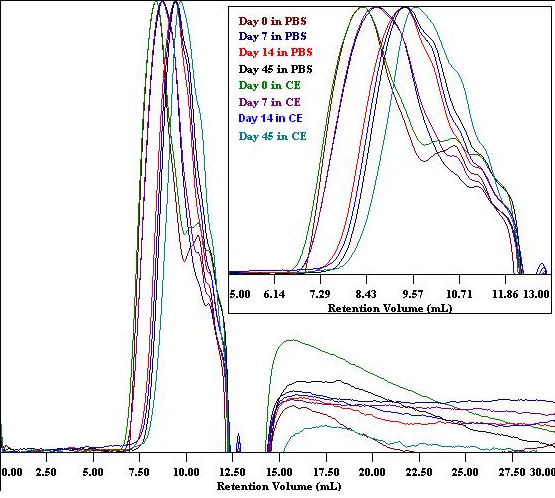
 **Fig. 10S:** GPC spectra for degradation of EPSHU in day 0, 7, 14 and 45 in both solutions PBS and CE.
